# Broad-Scale Assessment of Methylmercury in Adult Amphibians

**DOI:** 10.1021/acs.est.3c05549

**Published:** 2023-10-30

**Authors:** Brian J. Tornabene, Blake R. Hossack, Brian J. Halstead, Collin A. Eagles-Smith, Michael J. Adams, Adam R. Backlin, Adrianne B. Brand, Colleen S. Emery, Robert N. Fisher, Jill Fleming, Brad M. Glorioso, Daniel A. Grear, Evan H. Campbell Grant, Patrick M. Kleeman, David A. W. Miller, Erin Muths, Christopher A. Pearl, Jennifer C. Rowe, Caitlin T. Rumrill, J. Hardin Waddle, Megan E. Winzeler, Kelly L. Smalling

**Affiliations:** † U.S. Geological Survey, Northern Rocky Mountain Science Center, Missoula, Montana 59801, United States; ‡ Wildlife Biology Program, W. A. Franke College of Forestry & Conservation, University of Montana, Missoula, Montana 59812, United States; § U.S. Geological Survey, Western Ecological Research Center, Dixon, California 95620, United States; ∥ U.S. Geological Survey, Forest and Rangeland Ecosystem Science Center, Corvallis, Oregon 97331 United States; ⊥ U.S. Geological Survey, Western Ecological Research Center, San Diego, California 92101, United States; # U.S. Geological Survey, Eastern Ecological Science Center (Patuxent Wildlife Research Center), Turners Falls, Massachusetts 01376, United States; 7 U.S. Geological Survey, Wetland and Aquatic Research Center, Lafayette, Louisiana 70506, United States; 8 U.S. Geological Survey, National Wildlife Health Center, Madison, Wisconsin 53711, United States; 9 U.S. Geological Survey, Western Ecological Research Center, Point Reyes Station, California 94956, United States; 10 Department of Ecosystem Science and Management, Pennsylvania State University, University Park, Pennsylvania 16802, United States; 11 U.S. Geological Survey, Fort Collins Science Center, Fort Collins, Colorado 80526, United States; 12 U.S. Geological Survey, Wetland and Aquatic Research Center, Gainesville, Florida 32653, United States; 13 U.S. Geological Survey, New Jersey Water Science Center, Lawrenceville, New Jersey 08648, United States

**Keywords:** bioindicator, contaminant, ecotoxicology, frog, mercury, salamander

## Abstract

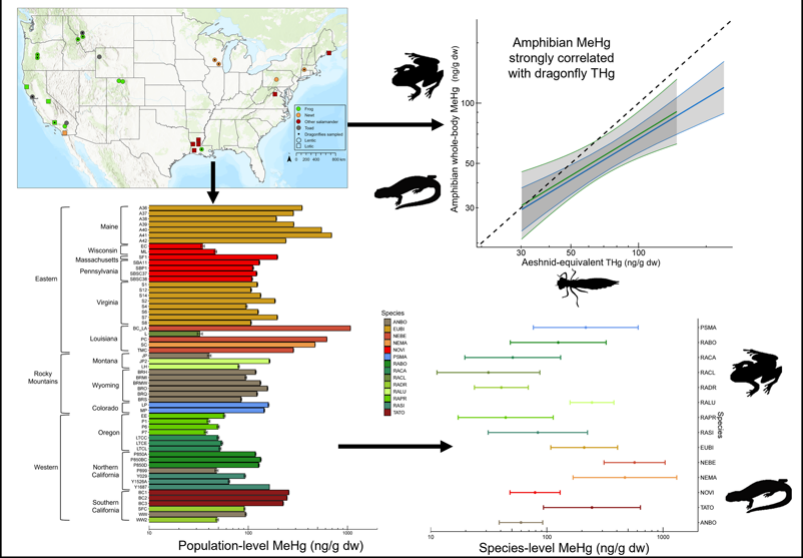

Mercury (Hg) is a toxic contaminant that has been mobilized and distributed worldwide and is a threat to many wildlife species. Amphibians are facing unprecedented global declines due to many threats including contaminants. While the biphasic life history of many amphibians creates a potential nexus for methylmercury (MeHg) exposure in aquatic habitats and subsequent health effects, the broad-scale distribution of MeHg exposure in amphibians remains unknown. We used nonlethal sampling to assess MeHg bioaccumulation in 3,241 juvenile and adult amphibians during 2017–2021. We sampled 26 populations (14 species) across 11 states in the United States, including several imperiled species that could not have been sampled by traditional lethal methods. We examined whether life history traits of species and whether the concentration of total mercury in sediment or dragonflies could be used as indicators of MeHg bioaccumulation in amphibians. Methylmercury contamination was widespread, with a 33-fold difference in concentrations across sites. Variation among years and clustered subsites was less than variation across sites. Life history characteristics such as size, sex, and whether the amphibian was a frog, toad, newt, or other salamander were the factors most strongly associated with bioaccumulation. Total Hg in dragonflies was a reliable indicator of bioaccumulation of MeHg in amphibians (R^2^ ≥ 0.67), whereas total Hg in sediment was not (R^2^ ≤ 0.04). Our study, the largest broad-scale assessment of MeHg bioaccumulation in amphibians, highlights methodological advances that allow for nonlethal sampling of rare species and reveals immense variation among species, life histories, and sites. Our findings can help identify sensitive populations and provide environmentally relevant concentrations for future studies to better quantify the potential threats of MeHg to amphibians.

## Abstract 2

We conducted the largest assessment of methylmercury bioaccumulation in adult amphibians. Methylmercury bioaccumulation was common and widespread but varied by site and life history characteristics.

## Introduction

Mercury (Hg) is a contaminant of global concern that can harm humans and wildlife.^1,2^ Although Hg occurs naturally, anthropogenic activities have increased environmental Hg concentrations several-fold higher than preindustrial background levels.^2^ In aquatic ecosystems, inorganic mercury can be microbially converted to methylmercury (MeHg),^2^ which increases Hg risks because MeHg is a more bioavailable and toxic form of Hg that also biomagnifies through food webs.^3^

Bioaccumulated MeHg can impair physiology, behavior, reproduction, and survival of wildlife^4−7^ through effects on endocrine, neurological, and immune systems.^8−10^ Most ecotoxicological research on effects of MeHg has focused on fishes, birds, and aquatic-dependent mammals, in part because fish consumption is one of the principal pathways for MeHg accumulation in wildlife and humans.^11^ However, Hg has also been shown to impair other species such as insectivores (e.g., bats and songbirds) at environmentally relevant exposures.^3^ Elevated MeHg exposure can also occur in other aquatic-dependent species, such as amphibians,^6,12,13^ which can result in effects such as reduced hatching success via egg infertility and embryonic mortality.^14^ However, in comparison to other aquatic-dependent species, relatively little is still known about the variability in MeHg bioaccumulation for most amphibians, which limits understanding of the toxicological effects and potential population-level threats from MeHg.^13,15^

Methylmercury may pose unique risks to amphibians because amphibians depend on aquatic environments associated with elevated MeHg production for key periods of their life history. Physiologically stressful events like metamorphosis and hibernation can also remobilize stored MeHg, redistribute tissue concentrations, and ultimately influence susceptibility to stressors, including emergent infectious diseases.^15^ Also, because many amphibians rely on aquatic and terrestrial environments, they can serve as MeHg vectors to both aquatic and terrestrial predators.^16,17^ Despite the potential threats that MeHg poses to amphibians, geographic variation in the magnitude, distribution, and variability of MeHg bioaccumulation among populations is not well documented in comparison to other taxa such as fishes and invertebrates.^3,18^ Most research on amphibian MeHg has focused on the aquatic, larval life stage rather than adults (but see refs (19−21)), despite adults generally occupying higher trophic positions and whose survival typically has a greater effect on population trajectories compared to larvae (e.g., refs (22 and 23)). Moreover, the imperiled status of many amphibian species precludes lethal sampling, and variation in tissues sampled across studies confound comparisons (e.g., nonlethal tissues, liver, whole-body).^24^ Finally, amphibian Hg concentrations are commonly measured for only total Hg (THg). Although data are limited on Hg speciation in amphibians,^12,18,19,25−27^ the reported proportion of Hg as MeHg is highly variable (e.g., MeHg:THg 7–109% for adults and metamorphic amphibians), emphasizing limitations of existing data.^12,19,28^

To provide insight into spatial variation in MeHg bioaccumulation, we sampled 3,241 juvenile and adult amphibians of 14 species from 26 populations across the conterminous USA. Nonlethal sampling of a single toe (frogs and toads) or small tail clip (salamanders) enabled determination of MeHg concentration for large numbers of animals, including for species of conservation concern. Our sampling represented a broad range of phylogenies and life history characteristics, allowing us to examine associations with species identity and traits, sex and size, and THg of sediment from sites where amphibians were captured. We also assessed the interannual variability in amphibian MeHg concentrations to evaluate the relative magnitude of temporal and spatial variation. Finally, we compared MeHg concentrations between amphibians and a geographically consistent bioindicator, dragonfly larvae,^18^ to provide a consistent index among sites where different amphibian taxa occurred and to evaluate the efficacy of dragonfly larvae as surrogates for cases where amphibians are rare or cannot be sampled.

## Materials and Methods

### Field Sites and Species

**Figure 1:**
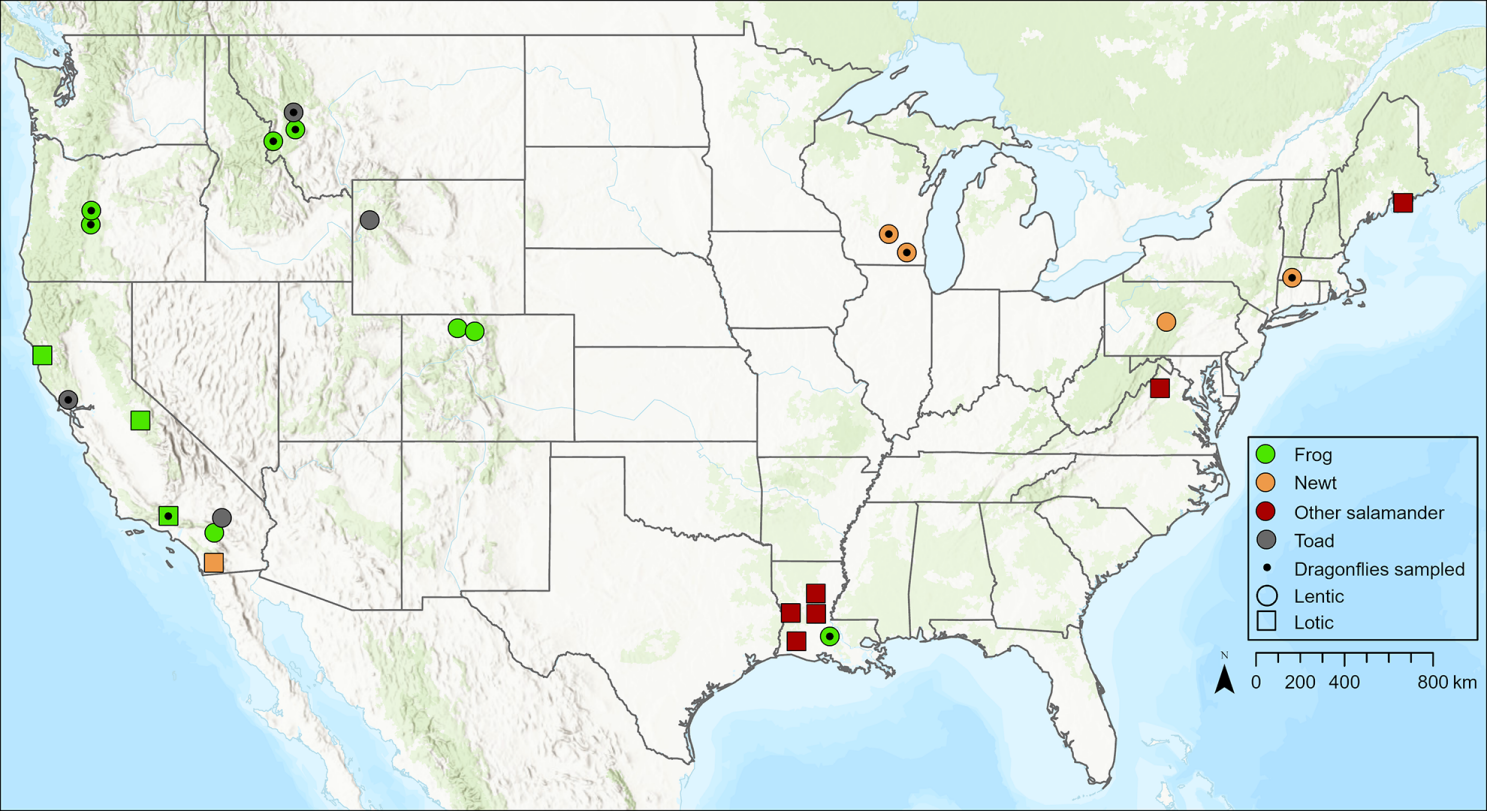
Sites sampled in the contiguous United States where we used nonlethal sampling to assess methylmercury bioaccumulation in adult amphibians. We collected a toe clip for frogs and toads and a tail tip for newts and other salamanders. Lentic sites are shown as circles, and lotic sites are shown as squares. We also collected dragonfly larvae from 10 sites and 13 unique subsites to measure the correlation between mercury accumulation in amphibians and dragonflies (point within circles or squares). Locations are jittered slightly to reduce overlap among sites. See Supplementary Table 1 for details on sites and species sampled. Note that dragonflies were sampled at a total of five separate subsites at two sites in Oregon, and dragonflies were collected for two species at one site in Montana; hence, only 11 points for dragonflies are displayed on the map (see Supplementary Table 7). Baselayer sources: Esri, Garmin, Food and Agriculture Organization, National Oceanic and Atmospheric Administration, U.S. Geological Survey, Environmental Protection Agency.

During 2017–2021, we sampled tissues from adult amphibians representing 14 species and 26 populations from 11 states across the United States (Figure 1 and Supplementary Tables 1 and 2). All animal procedures were reviewed and approved by the respective U.S. Geological Survey’s or the University’s Institutional Animal Care and Use Committee. Some sites were composed of neighboring wetlands or pools (i.e., we had 58 subsites nested within 24 sites). At two sites, we sampled two species; otherwise, only one species was sampled per site. We retained the subsite structure in analyses to examine the local variation in MeHg. Samples were primarily from adults (94% of samples) but also included some juveniles (6% of samples). Amphibian species sampled represented diverse life histories and included seven ranid frog species (*Rana* spp.), Boreal Chorus Frogs (*Pseudacris maculata*), Western Toads (*Anaxyrus boreas*), Eastern and California newts (*Notophthalmus viridescens* and *Taricha torosa*), Two-lined Salamanders (*Eurycea bislineata*), Mudpuppies (*Necturus maculosus*), and Gulf Coast Waterdogs (*N. beyeri*; Supplementary Tables 1 and 2). Four of seven ranid species are listed or proposed as threatened or endangered by the U.S. Fish and Wildlife Service: Sierra Nevada Yellow-legged Frog (*R. sierrae*; endangered), Oregon Spotted Frog (*R. pretiosa*; threatened), California Red-legged Frog (*R. draytonii*; threatened), and Foothill Yellow-legged Frog (*R. boylii*; proposed threatened or endangered).^29^ Most sites were temporary or permanent lentic sites (i.e., wetlands and ponds), and a few were lotic (i.e., streams; Figure 1).

### Sample Collection

We collected tissue samples from juvenile and adult amphibians from all 58 subsites within 24 sites between 2017 and 2021. Fifty-eight percent of sites were sampled in three or four consecutive years, whereas 42% were sampled in one or two years. Sites were selected to represent diverse geographic regions across the Unites States and generally associated with long-term monitoring studies (Supplementary Table 1).

We used dip nets, minnow traps, or hand captures to collect amphibians and held them in new plastic bags prior to processing. We measured the mass (g) and snout–vent length (mm) for each individual. We also collected a single toe from each frog or toad (generally posterior L4) or a small (∼2 cm) tail tip from each salamander;^30^ we only collected one sample from each individual. Toes were collected distal to the webbing, and tail clips were cut, using small disinfected scissors and placed in prelabeled polyethylene tubes with screwcaps. Following laboratory analysis, we converted toe or tail MeHg concentrations to whole-body MeHg concentrations using two regression equations derived from previous validation data (Equations S1 and S2).^31^ Following collection, all samples (amphibians, sediment, and dragonflies as described below) were held on ice until frozen at −20 °C and then shipped on dry ice to the laboratory for chemical analysis following established methods (see Supplementary Methods Laboratory Analyses). Briefly, samples were dried to a constant mass, and large samples (>50 mg dw) were homogenized to a fine powder in a porcelain mortar. Amphibian tissue samples were analyzed for MeHg following EPA method 1630, and THg concentrations in sediment and dragonfly larvae (see below) were analyzed following EPA method 7473.^32,33^

In 2020, we also collected dragonfly larvae from 10 sites and 13 unique subsites, where amphibians were being sampled to evaluate associations between Hg concentrations in both taxa. Prior comparisons between amphibian and dragonfly larvae indicated that THg concentrations in the two taxa were correlated, but comparisons were limited to a few amphibian species and only for THg in the amphibian tissues.^18^ Therefore, we sought to both expand the described relationship across a greater range of amphibian species with diverse life histories and evaluate relationships between amphibian MeHg and dragonfly larvae THg concentrations. We used dip nets to collect up to 15 dragonflies (mean = 11, range = 3–15; family Aeshnidae, Corduliidae, or Libellulidae) at each subsite, and samples were double-bagged in prelabeled, polyethylene zipper-seal bags. We quantified THg for dragonflies because ∼80% of Hg in dragonfly tissues is in the MeHg form, and THg concentrations are highly correlated (R^2^ = 0.96) with MeHg concentrations in dragonfly larvae.^18,34−36^

The primary goal of this effort was to evaluate the geographic and taxonomic distribution of MeHg exposure in amphibians across much of the US. Given this scope, we explicitly did not focus on measuring and quantifying mechanisms or processes associated with those exposure patterns. The one exception was the assessment of THg in sediments from the locations where amphibians were sampled. This aspect was implemented to evaluate whether bulk inorganic Hg contamination in substrate was associated with amphibian MeHg exposure, contrary to most findings in other aquatic organisms such as fish and invertebrates.^3^ Therefore, in 2019, we collected sediment from 30 subsites within 19 sites to evaluate associations between THg in sediment and MeHg of amphibians from the same sites. Within each subsite, we collected three sediment samples spaced ≥ 10 m apart in shallow water (<20 cm deep). With a gloved hand and plastic scoop, sediment was collected from the top 2 cm of surface sediment and placed in a clean polyethylene jar.

### Statistical Analyses

We used generalized linear mixed models (GLMM; package “lme4”^37^) to estimate how MeHg bioaccumulation in amphibians varied according to amphibian group (i.e., frog, toad, newt, or other salamanders), size (snout–vent length; hereafter, SVL), and sex (i.e., male, female, or unknown sex [unable to determine]). We included the amphibian group category because of distinct differences in larval period, foraging, and overwintering among species. “Frogs” included all ranid species and Boreal Chorus Frogs; “toads” included only Western Toads; “newts” included both Eastern and California newts; and “other salamanders” included Two-lined Salamanders, Mudpuppies, and Gulf Coast Waterdogs (i.e., plethodontids and proteids). To test whether relationships between MeHg and SVL were dependent on the amphibian group or sex, we used AIC to compare among three models: an additive model (group + SVL + sex), a model with an interaction between group and SVL (group × SVL + sex), and a model with an interaction between sex and SVL (group + SVL × sex). We did not include a fully interactive model (group × SVL × sex) or include interactions between SVL and site because there were no observations for certain combinations of the interactions and because of limited degrees of freedom, respectively. We included a random effect of subsites nested within sites and year (i.e., variation in MeHg related to differences among subsites, sites, and years). To account for variation in MeHg attributable to differences among species, we attempted to also include species, but models failed to converge because most sites included only a single species.

Most sites only contained a single target species, thus confounding the site with species in the analysis above. However, we sampled Eastern Newts and Western Toads from several locations (Figure 1), allowing for more detailed investigation of factors linked with MeHg bioaccumulation and providing a better assessment of variation among sites. Eastern Newts were sampled at four sites (seven subsites) in Massachusetts, Pennsylvania, and Wisconsin, and Western Toads were sampled at four sites (nine subsites) in California, Montana, and Wyoming. For both species, we used GLMMs to examine variation in MeHg bioaccumulation by including covariates of SVL and sex. To account for the variation in MeHg among years and subsites, we included these factors as random effects. We did not include a site random effect or nest subsites within sites because there were less than five sites.^38^ As mentioned above, we used AIC to compare between additive (SVL + sex) and interactive models (SVL × sex) to test whether relationships between MeHg and length were dependent on sex.

Amphibian MeHg concentrations were log_10_-transformed in all models. We calculated marginal and conditional R^2^ for all models; marginal R^2^ describes variance explained by fixed effects alone, and conditional R^2^ describes variance explained by fixed plus random effects (package “MuMIn”^39^). We also calculated variance inflation factors (VIFs) to assess multicollinearity among variables within models. Generally, VIFs less than ten are acceptable (Supplementary Table 3).^40,41^ To estimate mean MeHg concentrations for each of the 14 species, we used a univariate GLMM with species as the only predictor and included a random effect of subsites nested in sites, as described above. All statistical analyses were conducted, and assumptions of all analyses were checked, in Program R.^42^

We examined the relationships between THg in dragonfly larvae paired with amphibian MeHg (i.e., from the same sites) to test the efficacy of dragonfly larvae as bioindicators of MeHg bioaccumulation in amphibians (Figure 1). Concentrations of THg in dragonfly larvae were converted to Aeshnidae-equivalents^18^ to standardize across families. Next, we separately calculated geometric means for amphibian MeHg and dragonfly THg at each subsite. We used geometric means because of the skewness of the THg and MeHg data in both groups. We then used a linear regression to correlate site-specific geometric means of dragonfly THg with their paired amphibian MeHg geometric means for all amphibian species. To assess whether correlations between THg in dragonflies and MeHg in amphibians were similar across diverse life histories (i.e., all species) or more similar within closely related life histories, we used a separate linear regression correlating means for dragonfly THg and MeHg in *Rana* species only. The ranid species were the only taxa with sufficient data pairing dragonflies and amphibians (six ranid species from nine subsites).

We also examined the relationships between THg in sediment paired with amphibian MeHg (i.e., from the same sites) to determine the relationships between concentrations in sediment and amphibian tissues. As above, we calculated geometric means of amphibian MeHg at each site. We used a linear regression to correlate the geometric mean amphibian MeHg and sediment THg. Similar to our dragonfly analysis, we used a separate linear regression correlating means for sediment THg and MeHg in *Rana* species only.

## Results and Discussion

### Taxonomic and Geographic Variability

Mercury is a global contaminant of concern because of its vast distribution, toxicity, and propensity to biomagnify through food webs.^2^ However, there is still a lack of widespread information on environmental concentrations experienced by many organisms, especially for the more bioavailable and toxic form, MeHg.^2,43^ Across 3,241 juvenile and adult amphibians representing 14 different species from 26 populations (Figure 1 and Supplementary Tables 1 and 2), estimated whole body MeHg concentrations ranged from 8–4,197 ng/g dw, and the geometric mean was 86.0 ng/g dw (geometric standard deviation [GSD] = 2.07; interquartile range = 52.6–132.7 ng/g dw). There was six times greater variation in MeHg concentration among the 24 sites (geometric coefficient of variation [GCV] = 120%) than among the 58 subsites (GCV = 21%) or years within sites (GCV = 21%; Supplementary Figures 1 and 2).

**Figure 2:**
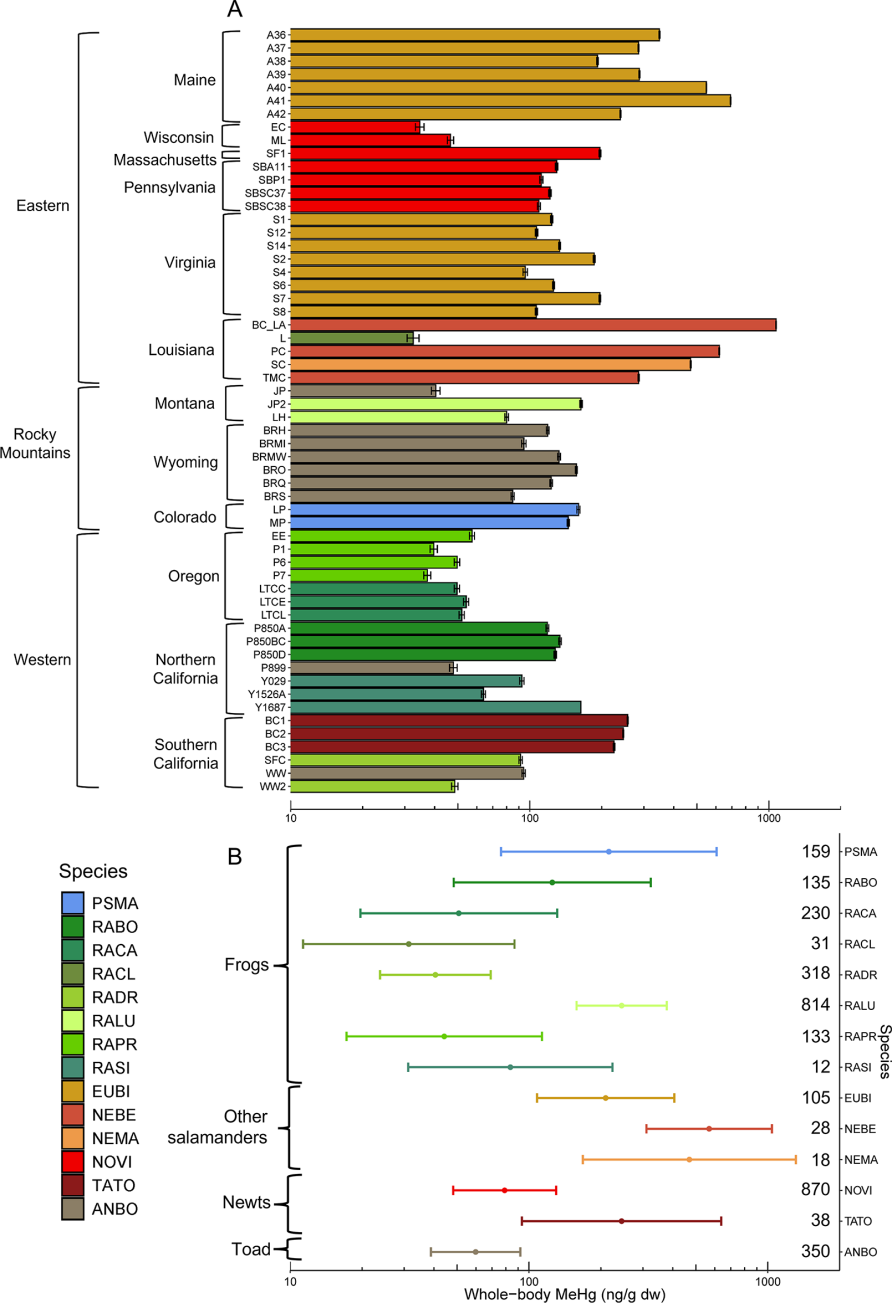
Methylmercury (MeHg) concentrations (ng/g dry weight [dw]) in adult amphibians collected across the contiguous United States. (A) Tops of bars (right side) represent geometric means (±geometric standard deviation). Colors represent species (See Supplementary Table 2 for species codes). Bars are generally ordered descending from the Eastern to the Western United States. (B) Model-estimated mean (and 95% confidence intervals) whole-body MeHg (ng/g dw) for each species. Numbers on the *y*-axis indicate sample sizes for each species. Colors in the legend indicate species in both panels (A and B): PSMA = Boreal Chorus Frogs (*Pseudacris maculata*), RABO = Foothill Yellow-legged Frog (*Rana boylii*), RACA = Cascades Frog (*Rana cascadae*), RACL = Green Frog (*Rana clamitans*), RADR = California Red-legged Frog (*Rana draytonii*), RALU = Columbia Spotted Frog (*Rana luteiventris*), RAPR = Oregon Spotted Frog (*Rana pretiosa*), RASI = Sierra Nevada Yellow-legged Frog (*Rana sierrae*), EUBI = Two-lined Salamander (*Eurycea bislineata*), NEBE = Gulf Coast Waterdog (*Necturus beyeri*), NEME = Mudpuppy (*Necturus maculosus*), NOVI = Eastern Newt (*Notophthalmus viridescens*), TATO = California Newt (*Taricha torosa*), ANBO = Western Toad (*Anaxyrus boreas*). Panel B shows how species were grouped (brackets) into broader amphibian groups—see Supplementary Table 2 for more information.

Among the species, we found a 33-fold difference in geometric mean concentrations. The lowest and highest geometric mean MeHg concentrations (±GSD) were for Green Frogs (*Rana clamitans*; 32.5 ± 1.9 ng/g dw) and Gulf Coast Waterdogs (1,071 ± 3 ng/g dw), respectively, in Louisiana (Figure 2A and Supplementary Table 5). Mudpuppies in Louisiana and Two-lined Salamanders in Maine also had some of the highest MeHg concentrations (geometric mean: 471 ± 2 and 691 ± 1 ng/g dw), whereas Western Toads in Montana, Oregon Spotted Frogs in Oregon, and California Red-legged Frogs in southern California had among the lowest geometric mean MeHg concentrations (40.4 ± 1.7, 37.3 ± 1.2, and 48.5 ± 1.5 ng/g dw; Figure 2A and Supplementary Table 5). Our results reveal that MeHg exposure is widespread in amphibians across the United States, including several threatened and endangered species. Some individuals had MeHg concentrations exceeding impairment benchmarks reported for fishes, as we describe below.^44^

The paucity of data for effects of MeHg on amphibians complicates estimating potential effects from MeHg bioaccumulation, particularly in the wild.^12,45^ Studies have identified negative effects of MeHg from coal ash residues and industrial manufacturing on amphibian larvae, but it is difficult to isolate effects of MeHg from other contaminants in these systems.^6,12,28,46−48^ There is substantial uncertainty in estimates of MeHg toxicity for adult amphibians, so for context, we evaluated exposure based on information from other aquatic species. In fishes, detrimental effects of MeHg can occur at concentrations ∼ 800 ng/g dw^44^ (assuming 75% water content of fishes and amphibians^49,50^). Around 95% of THg in fishes is MeHg, but similar to amphibians, this can vary by species, size, and life history.^51,52^ Around 0.5% of our individuals were above 800 ng/g dw.^44^ Resolving the uncertainty in effects of MeHg on adult amphibians is important for regulation and conservation, especially regarding the potential for interactions with other stressors, and determining benchmarks for amphibian MeHg exposure is an important future direction for research.

Previously reported MeHg concentrations in amphibians are comparable to measurements of similar species in our study (overall arithmetic mean = 116, SD = 144 ng/g dw). In four studies that measured MeHg in adult amphibians, site-level arithmetic mean concentrations ranged from ∼69 to 174 ng/g dw for Spotted Salamanders (*Ambystoma maculatum*) and ∼44 to 129 ng/g dw for Wood Frogs (*R. sylvatica*) across several sites in Vermont, USA.^21^ Arithmetic mean MeHg concentrations also ranged from ∼300 to 800 ng/g dw and 14 to 350 ng/g dw for American Bullfrogs (*R. catesbeianus*) in northern California and Arizona, USA, respectively.^19,20^ At a site contaminated by coal ash in Virginia, USA, arithmetic mean THg concentrations in adult Two-lined Salamanders ranged from ∼625 to 1,750 ng/g dw (assuming 50% MeHg: THg ratio; mean range 46–60% MeHg^53^) and was higher in aquatic Two-lined Salamanders than terrestrial Red-backed Salamanders (*Plethodon cinereus*, ∼300 ng/g dw MeHg assuming 50% MeHg: THg ratio).^12^ We did not target contaminated sites in our study, but concentrations for several species (e.g., Gulf Coast Waterdogs and Two-lined Salamanders) rivaled those of animals from contaminated sites sampled in a prior study.^12^

High variation in Hg among sites and species is common, yet proportional variation in our amphibian MeHg concentrations was lower than that reported for most other taxa. For example, amphibian MeHg in our study varied 33-fold, whereas THg concentrations varied ∼135-, 300-, and 496-fold among sites or species for dragonflies, birds, and fishes, respectively, in previous studies.^18,54,55^ These differences in magnitude and variation likely reflect the breadth of ecoregions, habitat types, and trophic diversity along with the size and age of animals sampled in the previous studies compared to ours (e.g., studies of birds and fish could compare long- and short-lived species or those with greater differences in size). We also found substantial variation between species within sites. Based on the only two sites where we sampled two species, it is clear that different species at a given site are not interchangeable; geometric mean (GSD) MeHg concentrations were ∼4 times lower for Western Toads (40.4 [1.7] ng/g dw) than Columbia Spotted Frogs (164 [1.8] ng/g dw) colocated in Montana, but concentrations were ∼2 times higher for Western Toads (94.2 [1.5] ng/g dw) than California Red-legged Frogs (48.5 [1.5] ng/g dw) co-occurring in southern California (Supplementary Figure 2). Western Toad MeHg concentrations were also ∼2 times higher for populations in California compared with those in Montana. Because adult Western Toads are mostly terrestrial, we expected their MeHg concentrations to be lower compared to more aquatic ranid frogs, similar to the contrast between aquatic Two-lined Salamanders and terrestrial Red-backed Salamanders in Virginia.^12^ However, observed patterns varied by location, suggesting factors such as biogeochemical influences on MeHg production and trophic pathways may have more influence on amphibian bioaccumulation^2,43^ than general habitat (aquatic vs terrestrial).

### Variation among Sizes, Sexes, and Groups of Amphibians

#### All Species

**Figure 3:**
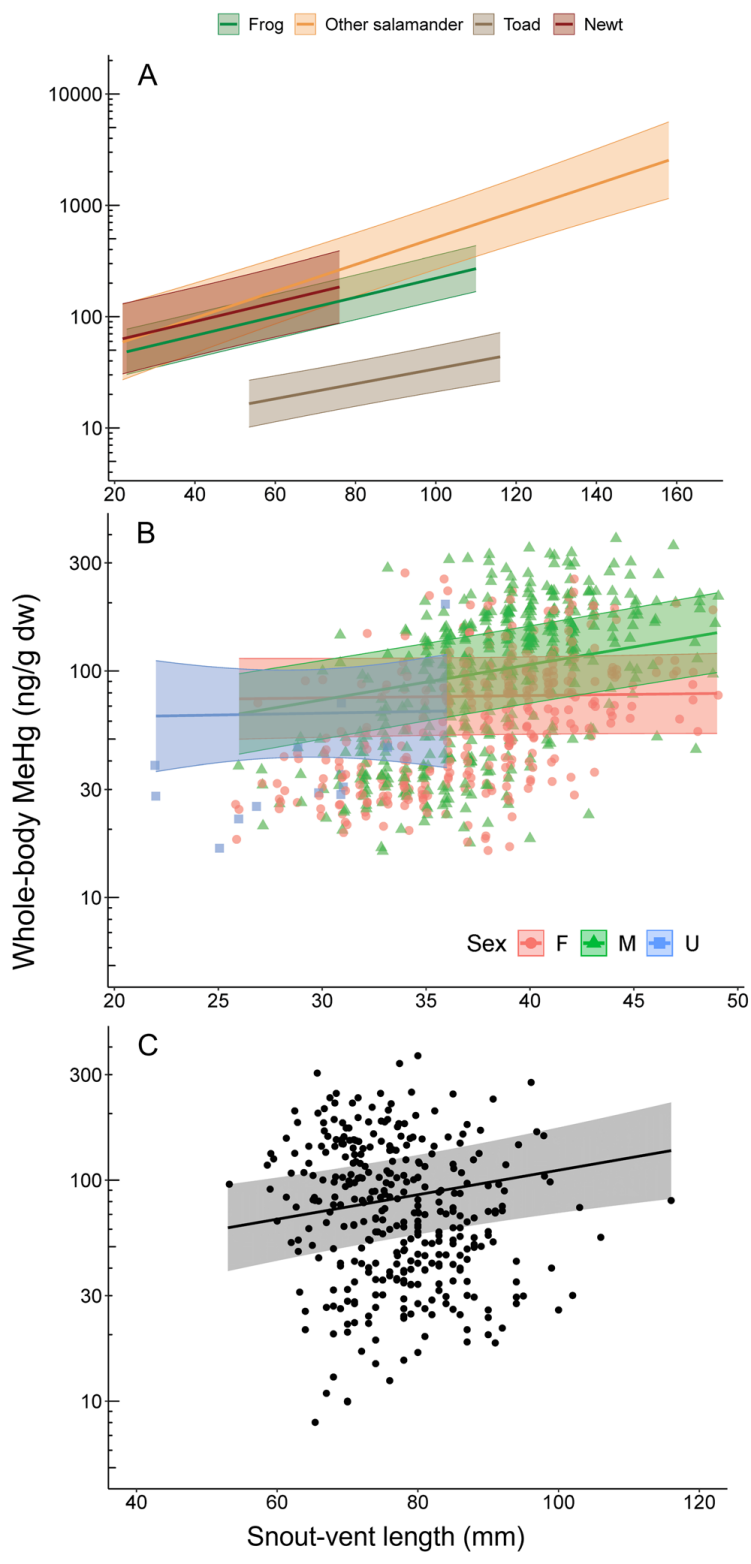
Relationships between model estimated (mean ±95% confidence intervals) whole-body methylmercury (MeHg; ng/g dry weight [dw]) and snout–vent length of amphibians (A) depended on the life history group (i.e., frog, other salamander [plethodontid or proteid], toad, or newt) when all species were included and (B) depended on sex for Eastern Newts alone (C) but did not depend on sex for Western Toads.

Characteristics such as size, sex, and whether the amphibian was a frog, toad, newt, or other salamander were strongly associated with MeHg bioaccumulation (Supplementary Tables 3 and 4). When comparing among additive and interactive models, the model with an interaction between the amphibian group and SVL (i.e., size) was most supported (ΔAIC ≥ 14.35; Supplementary Tables 3 and 4), indicating the relationship between MeHg and SVL depended on the group of amphibians more than the sex. Methylmercury increased with animal size; this bioaccumulation rate was higher in plethodontids and proteids (other salamanders; β = 0.003 ± 0.002 [standard error], *p* = 0.030) than in frogs, newts, or toads and lowest for toads compared to all other amphibian groups (β = −0.005 ± 0.001, *p* < 0.001; Figure 3 and Supplementary Table 4). There was also evidence that, for all species combined, males (β = 0.049 ± 0.008, *p* < 0.001) and individuals of unknown sex (β = 0.079 ± 0.017, *p* < 0.001) had higher MeHg than females, but confidence intervals for males and unknown sex overlapped with females (Supplementary Table 4 and Supplementary Figure 3). Based on previous studies, males and females may differ in their MeHg concentrations because of differences in behavior, physiology, or life history features, including maternal transfer from females to eggs.^56,57^

We were unable to fully separate variation based on species and site identity because the two were often confounded—we only sampled more than one species at two sites and had limited replication of species across sites. Based on the univariate model describing variation among species, plethodontids and proteids (other salamanders) generally had the highest MeHg concentrations, whereas several frogs (ranids) and Western Toads had lower MeHg, except for Columbia Spotted Frogs in Montana and Boreal Chorus Frogs in Colorado (Figure 2B and Supplementary Table 5). Across all species we sampled, mudpuppies and waterdogs sometimes feed on other aquatic vertebrates^58^ and likely had the highest trophic positions. The limited number of species with high trophic positions in our study limited our ability to assess relationships between trophic position or feeding ecology and MeHg bioaccumulation. Other salamanders may have had higher MeHg than frogs and toads because salamander larvae are predators whereas frog and toad larvae are primarily gazers,^59^ and adults may carry a greater MeHg load following metamorphosis.^21,60−62^ Indeed, this pattern was similar when Wood Frogs (*R. sylvatica*) were compared with Spotted Salamanders (*Ambystoma maculatum*) in Vermont (MeHg in adults) and when American Bullfrogs (*R. catesbeianus*) were compared to Two-lined Salamanders in Maine (THg in larvae).^21,62^ Both location and species identity, which include differences in size and life history, undoubtedly influence MeHg bioaccumulation. Given the current lack of information on drivers of variation in MeHg bioaccumulation in amphibians, future studies could emphasize sampling multiple species and life histories from the same sites to better control for variation.^54^ Future studies could also identify trophic position, possibly via stable isotopes (reviewed in ref (63)), and relate this to MeHg bioaccumulation.

#### Eastern Newts and Western Toads

We sought to better understand variation in MeHg concentrations among taxa by conducting separate analyses for the two species that were each sampled at four locations. The relationship between size (SVL) and MeHg in Eastern Newts depended on whether the newt was a male or female (support for the interactive model, SVL × sex; ΔAIC = 26.97). Bioaccumulation was greater for male newts (β = 0.015 ± 0.003, *p* < 0.001) than for unknown-sex newts (β = 0.001 ± 0.011, *p* = 0.961) or female newts (the reference level in the model), but confidence intervals were wide. The effect of sex on concentrations of MeHg in newts depended on SVL: at longer lengths, male newts had marginally higher MeHg than females, but this was opposite at shorter lengths (i.e., no difference, or females had marginally higher MeHg; Figure 3). For Western Toads, there was weak evidence that MeHg concentrations were lower in males than females (β = −0.059 ± 0.044, *p* = 0.187) and that the relationship between MeHg and SVL did not depend on sex (support for additive model, SVL + sex, ΔAIC = 1.77; Supplementary Table 4 and Supplementary Figure 3). It is unclear why we saw opposing trends for sex across all species compared to newts and toads separately (i.e., higher MeHg concentrations in females or in males, dependent on the set of species included). Life history characteristics vary widely in amphibians, which could account for some of this variation. For instance, Western Toads are long-lived compared to newts, and female toads are often much larger than males. Similar to our analysis with all species, size and sex in toads and newts were important predictors, but other geochemical factors also influence MeHg bioaccumulation.

### Assessing Dragonflies and Sediment as Indicators of Amphibian MeHg Exposure

**Figure 4:**
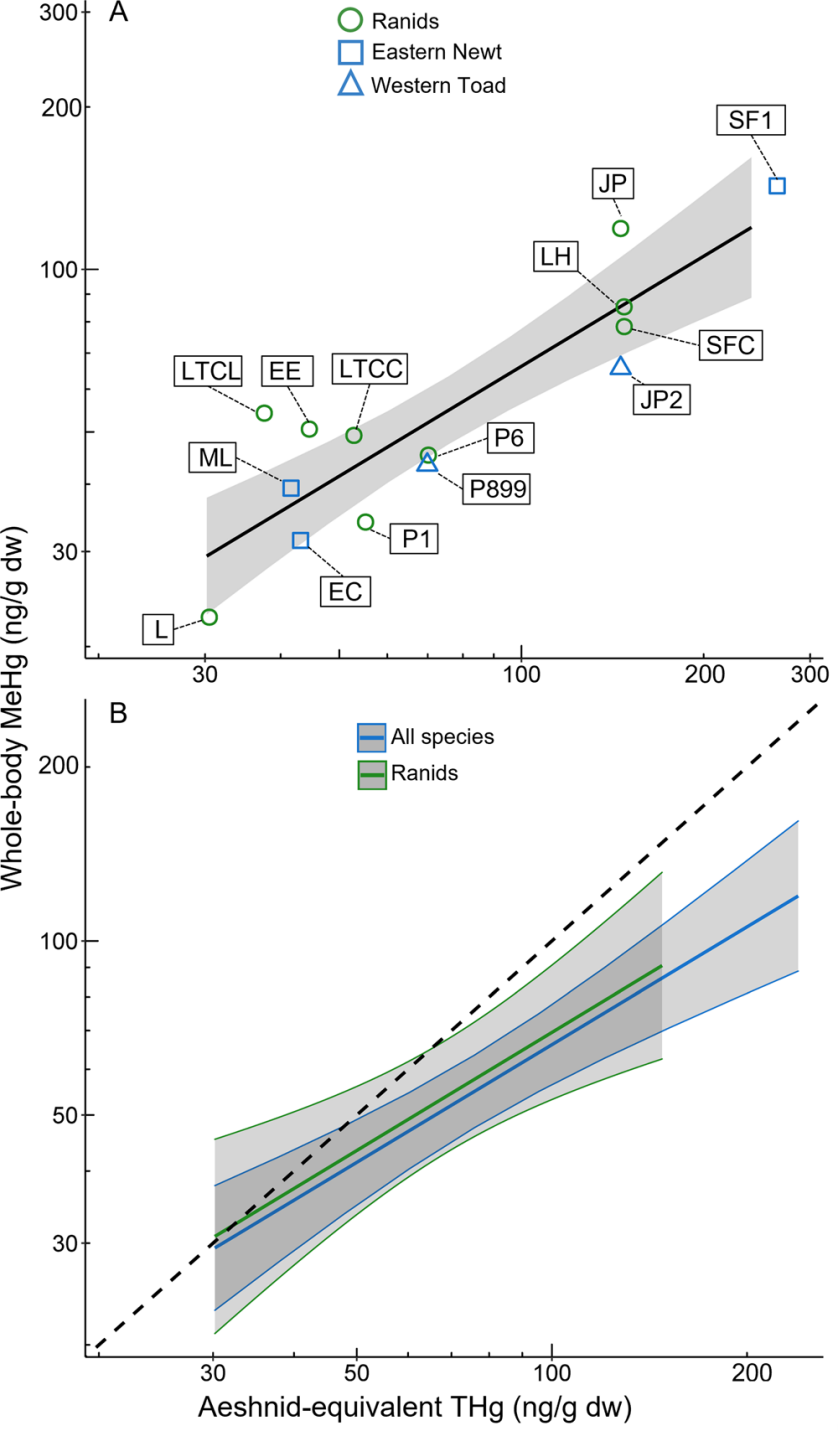
Associations between mean (±95% confidence interval) whole-body methylmercury (MeHg; ng/g dry weight [dw]) and aeshnid equivalent total mercury (THg, ng/g dw; after standardizing THg calculations among different dragonfly families using published equations) from amphibians and dragonflies collected from the same locations in 2020. Panel A includes all species and sites (see Figure 1 and Supplementary Table 1 for detailed site information), whereas panel B compares slopes for relationships between dragonfly THg and MeHg concentrations of all amphibian species and when only including ranid species. Both axes are log transformed. The dotted line represents a 1:1 relationship between amphibian MeHg and dragonfly aeshnid-equivalent THg.

Due to their predatory nature, ease of capture, and ubiquitous presence across waterbodies, dragonfly larvae can be effective sentinels for bioaccumulative contaminants like MeHg.^18^ Dragonfly THg concentrations were strongly correlated with MeHg in amphibian tissues for all species (β = 0.68 ± 0.10; F_1,12_ = 43.34; R^2^ = 0.76; *p* < 0.001) and for ranid species separately (β = 0.68 ± 0.16; F_1,7_ = 17.41; R^2^ = 0.67; *p* = 0.004; Figure 4, and Supplementary Table 6). We analyzed ranid frogs separately to better understand whether life history characteristics of amphibians influenced relationships between amphibian MeHg and dragonfly THg. Given that all amphibian species combined and the subset of ranids was similarly correlated with dragonfly THg, life history differences among amphibian species might not have a strong influence on the relationship between amphibian and dragonfly Hg bioaccumulation. The similarity in correlation among amphibian species sampled and dragonfly larvae in our study could also indicate that these amphibians all have similar feeding ecologies and thus a similar trophic position.

Total Hg concentrations in larval dragonflies have previously been shown to positively correlate with THg concentrations in a few amphibian species in the northwest and northeast United States (R^2^ ≤ 0.49),^18^ but our results from a wider range of species, life histories, and habitats nationwide were even more strongly correlated when comparing dragonfly THg to amphibian MeHg. To our knowledge, this is the first study to show that dragonfly THg is correlated with amphibian MeHg; this is important given the ratios of THg: MeHg in amphibians can vary immensely (7–109%) among species and life stages.^4,19,28,53,64,65^ The strong relationship between dragonfly THg and amphibian MeHg based on samples from the same sites suggests that dragonfly larvae can be an effective proxy for MeHg exposure, which may be especially important for rare species or those of conservation concern (e.g., threatened and endangered species).

Although sediments often represent long-term stores of Hg and can be sites of MeHg production, Hg concentrations in sediments are often decoupled from those in animals from the same site, perhaps because of the complex drivers of MeHg bioavailability and movement through food webs.^3,55,56^ Amphibian MeHg was not correlated with sediment THg across all species (β = −0.01 ± 0.15; *F*_1,28_= 0.01; R^2^ = −0.03; *p* = 0.917) or only ranid species (β = 0.16 ± 0.12; *F*_1,12_= 0.38; R^2^ = −0.04; *p* = 0.548; Supplementary Figure 4). Similarly, dragonfly THg was not correlated with sediment THg from the same sites (β = 0.08 ± 0.34; *F*_1,32_= 9.25; R^2^ = 0.004; *p* = 0.822). High variation in MeHg bioaccumulation among species and taxa has been demonstrated in our study and previous studies,^18,54,55^ and sediment THg is not a clear indicator of bioaccumulation or of risk to amphibians or other aquatic organisms.^66^ Future studies could investigate relationships between MeHg in porewater or bulk sediment, compared to sediment THg in the present study and MeHg in amphibians.

More precise environmental information, with a focus on areas where amphibians develop and spend most of their time, would be useful to understand the specific mechanisms of MeHg bioaccumulation. Although sampling animals that forage in the terrestrial environment and disperse among waterbodies (i.e., many juvenile and adult amphibians) limits our ability to test specific relationships and mechanisms of MeHg bioaccumulation, for most amphibian species, the juveniles and adults contribute more to population growth than larvae and are thus most important when assessing population risk.^22,23^ Additionally, other biogeochemical factors such as dissolved organic carbon, terminal electron acceptors, and redox can influence mercury speciation and methylation and could inform potential for MeHg bioaccumulation.^67−69^ Although we included a large number of sites in this study, increasing the number of sites sampled would also contribute to a better understanding of how environmental characteristics affect MeHg production and bioaccumulation.

Our results provide insight into the range of MeHg concentrations in amphibians across the United States, with concentrations ranging widely from 8 to 4,197 ng/g dw. Based on nonlethal methods that allowed us to sample several imperiled and protected species, our study represents the largest assessment of MeHg bioaccumulation in amphibians based on geographic scope, number of species sampled, and sampling intensity. Across our broad geographic scope, MeHg bioaccumulation in adults varied by sex, size, species, and life history, but specific factors associated with MeHg bioaccumulation remain unresolved. Determining these factors could best be accomplished by sampling different life stages of several species at multiple sites across a broad geographic range,^60^ coupled with detailed information on trophic position and other mechanistic processes affecting MeHg bioaccumulation. It is also important to conduct studies to translate observed MeHg concentrations into measures of risk for sublethal and lethal effects on amphibians, particularly adults, under field conditions. Similarly, for cases in which amphibians are rare or cannot be sampled, our results suggest that dragonfly larvae reflect MeHg bioaccumulation of amphibians from the same sites, whereas sediment is an ineffective indicator of bioaccumulation or risk of exposure. Finally, our findings can inform environmentally relevant MeHg concentrations for future exposure studies and help identify sensitive or at-risk populations.

## Supporting Information Available

Supplementary laboratory analyses methods. Table S1 summary of sites where samples were collected; Table S2 summary of samples by species and groups of amphibians; Table S3 AIC table and description of mixed-effects regression models; Table S4 summary statistics from mixed-effects regression models; Table S5 summary statistics from mixed-effects regression model for species differences; Table S6 summary of amphibian and dragonfly tissue samples collected at the same sites. Figure S1 variation in whole body methylmercury for subsites within sites; Figure S2 variation in whole body methylmercury among years within sites; Figure S3 relationship between whole-body methylmercury and sex; Figure S4 relationships between sediment THg and whole-body amphibian MeHg. Equations S1 and S2: regression equations describing relationships between amphibian toe or tail clip and whole-body methylmercury (PDF)
