# Supplementary material for: Broad-Scale Assessment of Methylmercury in Adult Amphibians
Source: Environ Sci Technol. 2023 Oct 30;57(45):17511–21. doi: 10.1021/acs.est.3c05549 (PMC10653216; doi:10.1021/acs.est.3c05549)
Supplement: Supplementary file 1 — es3c05549_si_001.pdf [file es3c05549_si_001.pdf]

Broad-scale Assessment of Methylmercury in Adult Amphibians

SUPPORTING INFORMATION

Brian J. Tornabene[1], Blake R. Hossack[1,2], Brian J. Halstead[3], Collin A. Eagles-Smith[4], Michael J. Adams[4], Adam R. Backlin[5], Adrianne B. Brand[6], Colleen S. Emery[4], Robert N. Fisher[5], Jill Fleming[6], Brad M. Glorioso[7], Daniel A. Grear[8], Evan H. Campbell Grant[6], Patrick M. Kleeman[9], David A.W. Miller[10], Erin Muths[11], Christopher A. Pearl[4], Jennifer C. Rowe[4], Caitlin T. Rumrill[4], J. Hardin Waddle[12], Megan E. Winzeler[8], and Kelly L. Smalling[13]

[1] U.S. Geological Survey, Northern Rocky Mountain Science Center, Missoula, MT 59801, USA
[2]Wildlife Biology Program, W. A. Franke College of Forestry & Conservation, University of Montana, Missoula, Montana, USA
[3]U.S. Geological Survey, Western Ecological Research Center, Dixon, CA 95620, USA
[4]U.S. Geological Survey, Forest and Rangeland Ecosystem Science Center, Corvallis, OR 97331 USA
[5]U.S. Geological Survey, Western Ecological Research Center, San Diego, CA 92101, USA
[6]U.S. Geological Survey, Eastern Ecological Science Center, Turners Falls, MA 01376, USA
[7]U.S. Geological Survey, Wetland and Aquatic Research Center, Lafayette, LA 70506 USA
[8]U.S. Geological Survey, National Wildlife Health Center, Madison, WI 53711, USA
[9]U.S. Geological Survey, Western Ecological Research Center, Point Reyes Station, CA 94956, USA
[10]Department of Ecosystem Science and Management, Pennsylvania State University, University Park, PA 16802, USA
[11]U.S. Geological Survey, Fort Collins Science Center, Fort Collins, CO 80526, USA
[12]U.S. Geological Survey, Wetland and Aquatic Research Center, Gainesville, FL 32653, USA
[13]U.S. Geological Survey, New Jersey Water Science Center, Lawrenceville, NJ 08648 USA

14 Pages
Supplementary Methods: Laboratory Analyses
6 Tables
4 Figures
2 Equations

METHODS

Laboratory Analyses

Amphibian tissue clips were freeze dried and dragonfly larvae were oven dried at 50°C for 48–96 hours until a constant mass was achieved. Tail clips and dragonfly larvae with a dry mass >30 mg were homogenized to a fine powder using stainless steel scissors and glass rods. All toe clips and other samples with a mass < 30 mg were analyzed whole. Methylmercury concentrations were measured on all toe and tail clips following EPA method 1630 (U.S. Environmental Protection Agency, 2001) at the USGS Forest and Rangeland Ecosystem Science Center.

Briefly, entire tissue clips (or approximately 10 mg of dried, homogenized sample) were digested in 4 ml of 30% nitric acid at 60◦C overnight (~15 hours) and then ethylated with 1% sodium tetra-ethylborate prior to being analyzed via cold-vapor atomic fluorescence spectrometry on a MERX-M (Brooks Rand Instruments, Seattle, Washington, USA) automated methylmercury analyzer. Digests of toe clip samples weighing < 1 mg were analyzed in triplicate and the average of the triplicate values was used for those samples. Quality assurance measures included analysis of two independently derived liquid calibration standards, two certified reference materials (either scallop tissue [IAEA-452; International Atomic Energy Agency, Vienna, Austria], DORM-4, or TORT-3), reagent blanks, a matrix spike, and sample duplicates with every run of 72 samples. Average (± standard error) percent recoveries were 100.0 ± 0.3% and 100.8 ± 0.2% for liquid standards (n = 579), and certified reference materials (n = 1276), respectively. Matrix spike recoveries for MeHg averaged 106.1 ± 0.6% (n = 276) and the mean relative percent difference for all duplicates was 4.0 ± 0.3% (n = 162). Method blanks for MeHg ranged from < 0.01 to 0.58 pg with a median of 0.03 pg (n = 986). For dragonfly larvae, whole bodies were analyzed in larvae < 30 mg dw, whereas a 20–30 mg aliquot of the homogenate was used for analysis of larger individuals. Total mercury concentrations were determined at the USGS Contaminant Ecology Research Lab (CERL) using either a Milestone DMA-80 (Milestone Inc., Monroe, Connecticut, USA) or Nippon MA-3000 (Nippon Instrument Corporation, Osaka, Japan) Hg analyzer following EPA method 7473 (U.S. Environmental Protection Agency, 2000). Quality assurance measures for THg included analysis of two certified reference materials (either dogfish muscle tissue [DORM-4; National Research Council of Canada, Ottawa, Canada], dogfish liver [DOLT-3; National Research Council of Canada, Ottawa, Canada], lobster hepatopancreas [TORT-3; National Research Council of Canada, Ottawa, Canada], or pine needles [IAEA 1575A; International Atomic Energy Agency, Vienna, Austria]), two continuing calibration checks, two system and method blanks, and two sample duplicates at least every 10 samples. Recoveries (± standard deviation) averaged 98.8 ± 0.75% (n = 44) and 106.9 ± 2.2% (n = 34) for certified reference materials and calibration checks, respectively. The absolute relative percent difference for all duplicates averaged 4.28 ± 1.6%.

TABLES

Supplementary Table 1. Summary of states, sites, subsites, species, and number of tissue samples collected from amphibian species throughout the contiguous United States. The bottom row (Total) describes the number of unique states, sites, subsites, and species samples were collected from while the total for samples describes the total number of samples included in our study. The asterisk (*) indicates that the sum of the column is 26 total sites, but two separate species were sampled at two sites such that the total is 24 unique sites. See Supplementary Table 1 for more information.

	State	Site	Subsites	Species	Common name	Samples
1	Wisconsin	Emma Carlin	EC	*Notophthalmus viridescens*	Eastern Newt	215
2	Wisconsin	Mud Lake	ML	*Notophthalmus viridescens*	Eastern Newt	137
3	Pennsylvania	Scotia Barrens	SBA11, SBP1, SBSC37, SBSC38	*Notophthalmus viridescens*	Eastern Newt	461
4	Massachusetts	Springfield	SF1	*Notophthalmus viridescens*	Eastern Newt	57
5	Maine	Acadia	A36, A37, A38, A39, A40, A41, A42	*Eurycea bislineata*	Two-lined Salamander	52
6	Virginia	Shenandoah	S1, S2, S4, S6, S7, S8, S12, S14	*Eurycea bislineata*	Two-lined Salamander	53
7	Oregon	Dillman Meadows	P1, P6, P7, EE	*Rana pretiosa*	Oregon Spotted Frog	133
8	Oregon	Little Three Creeks	LTCC, LTCE, LTCL	*Rana cascadae*	Cascades Frog	230
9	Montana	Jones Pond	JPA	*Anaxyrus boreas*	Western Toad	108
10	Montana	Jones Pond	JPR	*Rana luteiventris*	Columbia Spotted Frog	315
11	Montana	Lost Horse	LH	*Rana luteiventris*	Columbia Spotted Frog	499
12	Wyoming	Black Rock	BRH, BRMI, BRMW, BRO, BRQ, BRS	*Anaxyrus boreas*	Western Toad	165
13	Colorado	Lily Pond	LP	*Pseudacris maculata*	Boreal Chorus Frog	83
14	Colorado	Matthews Pond	MP	*Pseudacris maculata*	Boreal Chorus Frog	76
15	Louisiana	Labyrinth	L	*Rana clamitans*	Green Frog	31
16	Louisiana	Brushy Creek	BC_LA	*Necturus beyeri*	Gulf Coast Waterdog	6
17	Louisiana	Prairie Creek	PC	*Necturus beyeri*	Gulf Coast Waterdog	12
18	Louisiana	Spring Creek	SC	*Necturus maculosus*	Mudpuppy	18
19	Louisiana	Ten Mile Creek	TC	*Necturus beyeri*	Gulf Coast Waterdog	10
20	California	Fox Creek	P850A, P850BC, P850D	*Rana boylii*	Foothill Yellow-legged Frog	135
21	California	Sonoma Mountain	P899	*Anaxyrus boreas*	Western Toad	74
22	California	Summit Meadow	Y1526A, Y1687	*Rana sierrae*	Sierra Nevada Yellow-legged Frog	12
23	California	Boulder Creek	BC1, BC2, BC3	*Taricha torosa*	California Newt	38

24	California	Sanfrancisquito Creek	SFC	*Rana draytonii*	California Red-legged Frog	308
25	California	Whitewater	WWA	*Anaxyrus boreas*	Western Toad	3
26	California	Whitewater	WWR	*Rana draytonii*	California Red-legged Frog	10
**Total**	11	24*	58	14	14	3241

Supplementary Table 2. Summary of species, samples, and sites where tissues (toes or tails) were collected from amphibians to evaluate methylmercury bioaccumulation. Fourteen species of amphibians (eight frog, five salamander, two newts, and one toad species) were included in our study ordered from highest to lowest number of samples. Most species were generally sampled at small geographic scales (e.g., at one site or several sites in one state), but Eastern Newts and Western Toads were broadly sampled across the Eastern and Western United States, respectively (see Figure 1). Group describes which amphibian group we categorized them as including frog, newt, other salamander (plethodontid or proteid), or toad. Code is the species code used in tables and figures. The asterisk (*) indicates that the sum of the column is 26 total sites, but two separate species were sampled at two sites such that the total is 24 unique sites. See Supplementary Table 1 for more information.

**Species**	**Group**	**Code**	**Samples**	**Sites**
Eastern Newt	Newt	NOVI	870	4
Columbia Spotted Frog	Frog	RALU	814	2
Western Toad	Toad	ANBO	350	4
California Red-legged Frog	Frog	RADR	318	2
Cascades Frog	Frog	RACA	230	1
Boreal Chorus Frog	Frog	PSMA	159	2
Foothill Yellow-legged Frog	Frog	RABO	135	1
Oregon Spotted Frog	Frog	RAPR	133	1
Two-lined Salamander	Other salamander	EUBI	105	2
California Newt	Newt	TATO	38	1
Green Frog	Frog	RACL	31	1
Gulf Coast Waterdog	Other salamander	NEBE	28	3
Mudpuppy	Other salamander	NEMA	18	1
Sierra Nevada Yellow-legged Frog	Frog	RASI	12	1
**Total**		14	3241	24*

Supplementary Table 3. Mixed-effects regression models, explanatory variables, random effects included in each model, and model selection criteria to evaluate variation in whole-body methylmercury accumulation in amphibians from throughout the contiguous United States. We tested among additive and interactive models for each of the models listed (all species and subsets) to test whether relationships between MeHg and snout-vent length (SVL) depended on group of amphibians (F = frog, N = newt, T = toad, S = salamander) for all species or depended on sex for when Western Toads and Eastern Newts were subsetted. Methylmercury data were $\log_{10}$ transformed to account for the skewness common to ecotoxicological data. For the All-Species models, group compares among frogs, newts, toads, and other salamanders (i.e., plethodontids and proteids). Habitat type is lotic or lentic and was not included in the subset model for Western Toads because they were only sampled in lentic habitats. See text for other model specifics. Temperature and precipitation are 10 y means from 1 km pixels around each site taken from DAYMET (see text for full details). LOI = loss of ignition, a measure of organic matter in sediment collected at sites. For random effects, we nested subsites within sites and accounted for variation explained by years in All-Species models and only included a random effect of year in subset models (see text for full details). LL = log likelihood, AIC = Akaike Information Criterion, ΔAIC = difference in AIC from top model, R^2m = marginal R-squared (variation described by fixed effects alone), R^2c = conditional R-squared (variation described by fixed and random effects), and VIF = variance inflation factor.

**Model**	**Predictors**	**LL**	**AIC**	**ΔAIC**	**R^2m**	**R^2c**
All Species	Group (F, N, T, S) × SVL + Sex	954.03	-1880.06	0.00	0.12	0.89
	Group (F, N, T, S) + SVL + Sex	943.86	-1865.71	14.35	0.12	0.87
	Group (F, N, T, S) + Sex × SVL	945.25	-1864.49	15.57	0.12	0.87
Subset: Western Toads	SVL + Sex	39.98	-67.97	0.00	0.56	0.60
	SVL × Sex	40.10	-66.20	1.77	0.04	0.60
Subset: Eastern Newts	SVL × Sex	320.92	-623.84	0.00	0.06	0.69
	SVL + Sex	305.44	-596.87	26.97	0.05	0.67

Supplementary Table 4. Summary statistics from mixed-effects regression models evaluating accumulation of methylmercury in all species of amphibians, only Western Toads, and only Eastern Newts (‘Model’) collected from the contiguous United States. SE = standard error. See Table 2 for model selection statistics. Sex estimates represent differences from females and group estimates represent differences from frogs—the reference levels for these categorical variables. The reference level for habitat type (‘type’) is lotic and for hydroperiod is ephemeral. Loss of ignition represents percent of organic matter in sediment.

**Model**	**Variable**	**β**	**SE**	***t***	***p***
All Species	Intercept	1.292	0.156	8.29	< 0.001
	Group: Newt	0.311	0.336	0.93	0.368
	Group: Other salamander	0.079	0.313	0.25	0.802
	Group: Toad	0.063	0.391	0.16	0.875
	SVL	0.010	0.001	18.06	< 0.001
	Sex: Male	0.049	0.008	6.06	< 0.001
	Sex: Unknown	0.079	0.017	4.56	< 0.001
	Group: Newt x SVL	-0.001	0.002	-0.45	0.656
	Group: Other salamander x SVL	0.003	0.002	2.18	0.030
	Group: Toad x SVL	-0.005	0.001	-3.71	< 0.001
Subset: Western Toad	Intercept	1.543	0.174	8.85	< 0.001
	SVL	0.006	0.002	3.37	0.001
	Sex: Male	-0.059	0.044	-1.32	0.187
Subset: Eastern Newt	Intercept	1.850	0.120	15.48	< 0.001
	SVL	0.001	0.002	0.485	0.627
	Sex: Male	-0.445	0.099	-4.485	< 0.001
	Sex: Unknown	-0.084	0.335	-0.252	0.801
	SVL x Sex: Male	0.015	0.003	5.584	< 0.001
	SVL x Sex: Unknown	0.001	0.011	0.049	0.961

Supplementary Table 5. Summary statistics from mixed-effects regression models evaluating differences in accumulation of methylmercury in different species of amphibians collected from the contiguous United States. SE = standard error. Estimates represent differences from Western Toads, the reference level in the model.

**Variable**	**β**	**SE**	***t***	***p***
Intercept	1.78	0.10	18.61	< 0.001
Two-lined Salamander	0.54	0.17	3.11	0.006
Gulf Coast Waterdog	0.98	0.16	5.94	< 0.001
Mudpuppy	0.89	0.25	3.63	0.001
Eastern Newt	0.12	0.15	0.83	0.413
Boreal Chorus Frog	0.56	0.25	2.24	0.034
Foothill Yellow-legged Frog	0.32	0.23	1.39	0.182
Cascades Frog	-0.07	0.23	-0.31	0.763
Green Frog	-0.28	0.25	-1.14	0.265
California Red-legged Frog	-0.17	0.11	-1.59	0.113
Columbia Spotted Frog	0.61	0.02	29.85	< 0.001
Oregon Spotted Frog	-0.13	0.23	-0.57	0.574
Sierra Nevada Yellow-legged Frog	0.15	0.24	0.61	0.548
California Newt	0.61	0.23	2.62	0.017

Supplementary Table 6. Summary of amphibian tissue and dragonfly samples collected at the same location for thirteen unique subsites in the United States in 2020. Two separate species were collected at the same site in Montana (Jones Pond). Common name describes the amphibian species tissues were collected from, count describes the number of samples collected for amphibians or dragonflies, and SD = standard deviation.

			Amphibian tissue			Dragonfly		
**State**	**Subsite**	**Common name**	**Count**	**Geometric mean**	**Geometric SD**	**Count**	**Geometric mean**	**Geometric SD**
Wisconsin	Emma Carlin	Eastern Newt	80	31.62	1.25	15	43.15	1.50
Wisconsin	Mud Lake	Eastern Newt	40	39.33	1.08	8	41.60	1.32
Massachusetts	Springfield	Eastern Newt	10	142.85	1.25	15	264.58	2.18
Oregon	East Excavation	Oregon Spotted Frog	8	50.61	1.16	15	44.61	1.48
Oregon	Pond 1	Oregon Spotted Frog	14	34.03	1.23	12	55.25	1.31
Oregon	Pond 6	Oregon Spotted Frog	9	45.29	1.50	15	70.17	1.29
Oregon	LTCC	Cascades Frog	19	48.73	1.42	3	52.86	1.22
Oregon	LTCL	Cascades Frog	8	54.17	1.51	11	37.59	1.48
Montana	Jones Pond 1	Western Toad	9	65.64	1.18	7	145.87	1.71
Montana	Jones Pond 2	Columbia Spotted Frog	93	119.07	1.18	7	145.87	1.45
Montana	Lost Horse	Columbia Spotted Frog	154	85.28	1.30	10	147.78	1.37
Louisiana	Labyrinth	Green Frog	15	22.67	1.22	15	30.49	1.53
California	P899	Western Toad	15	43.45	1.40	15	69.88	1.60
California	San Francisquito	California Red-legged Frog	61	78.38	1.36	14	147.86	1.45

FIGURES

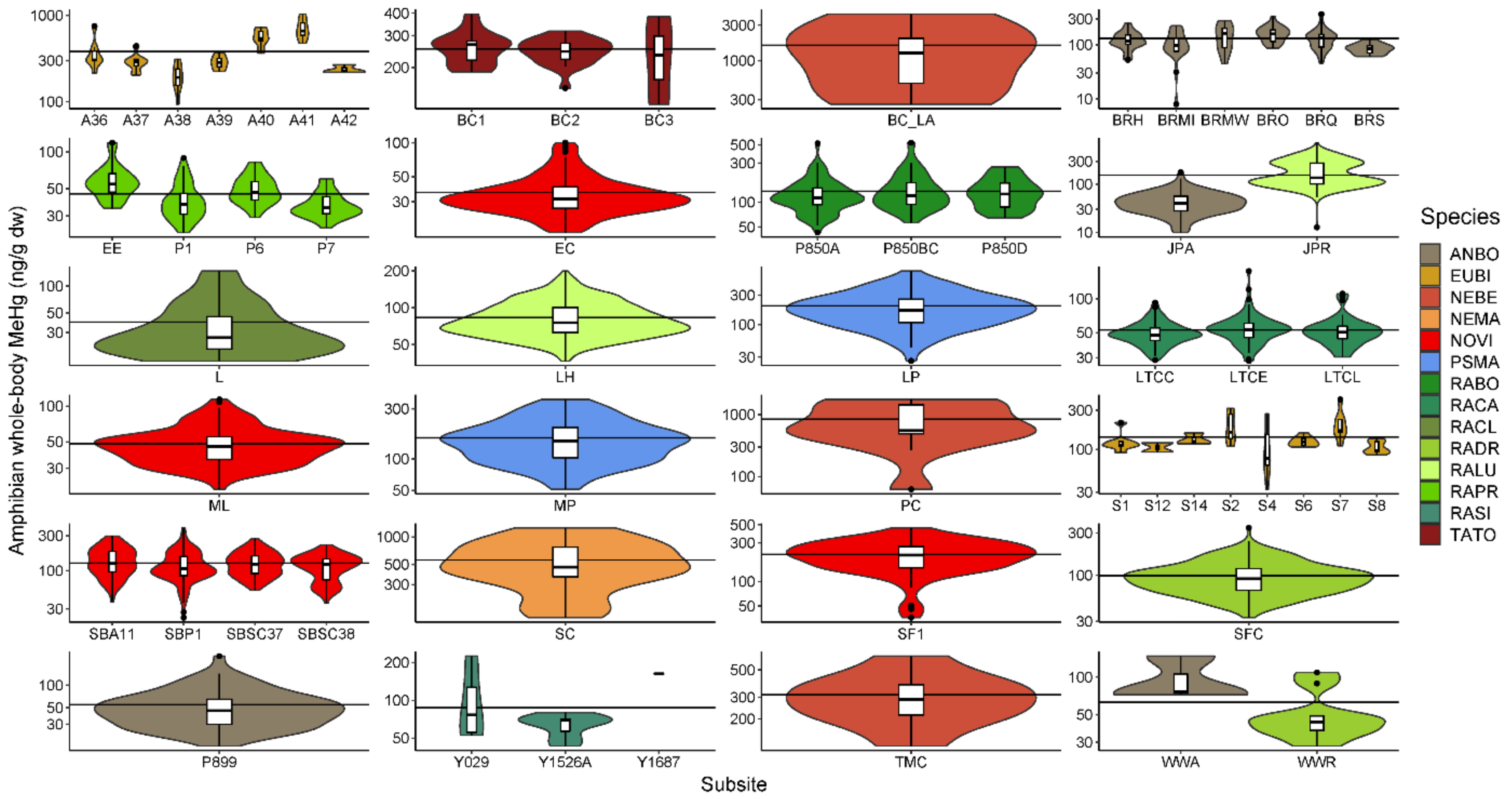

Supplementary Figure 1. Variation in amphibian whole body methylmercury (MeHg; ng/g dry weight [dw]) bioaccumulation for subsites within sites. The horizontal line in each facet is the site grand mean for subsites within that site. Note different scales of y-axes in each facet.

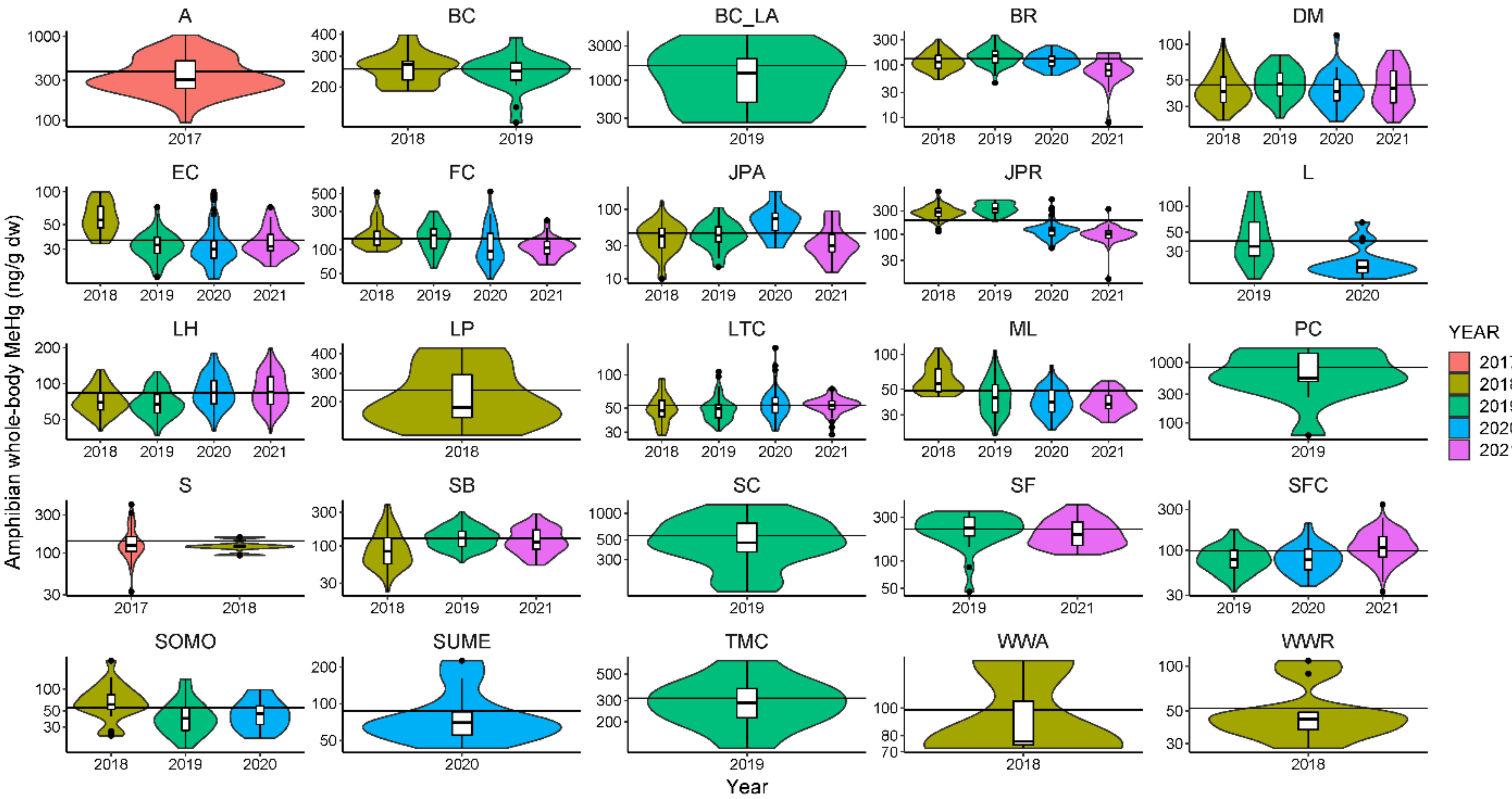

Supplementary Figure 2. Annual variation in amphibian whole body methylmercury bioaccumulation (MeHg; ng/g dry weight [dw]) within sites. Some sites only had one year of sampling whereas other sites had several years of sampling. The horizontal line in each facet is the site grand mean of years when samples were collected within that site.

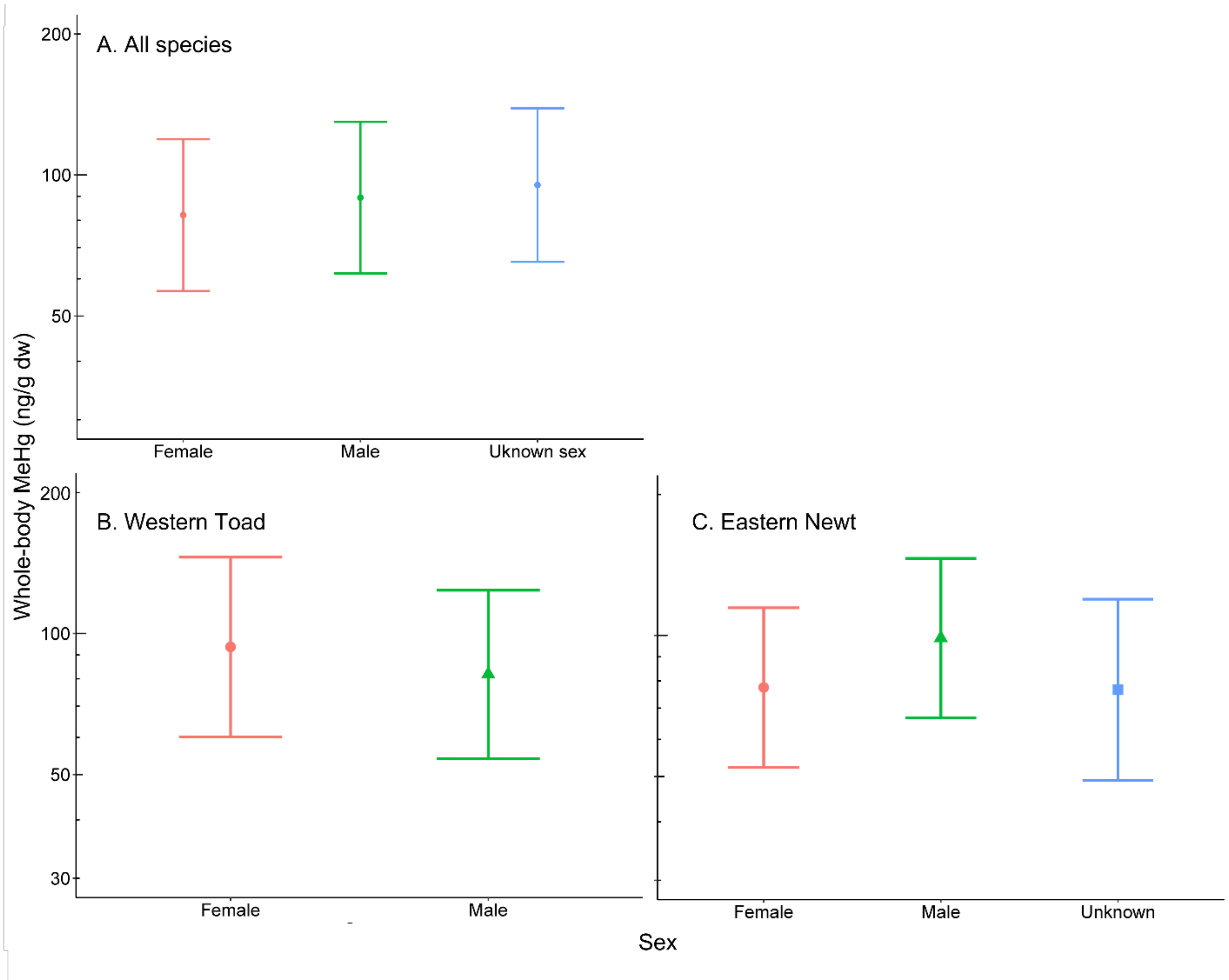

Supplementary Figure 3. Relationship between model-estimated (mean ± 95% confidence interval) whole-body methylmercury (MeHg; ng/g dry weight [dw]) and sex for (A) all amphibians, (B) Western Toads, and (C) Eastern Newts sampled from across the contiguous United States. In plate C, this represents at larger snout-vent lengths given that the relationship between length and MeHg was dependent on sex (females have higher MeHg than males at lower SVL). In plate C, there was no interaction between snout-vent length and sex and thus represents differences in sex across all snout-vent lengths.

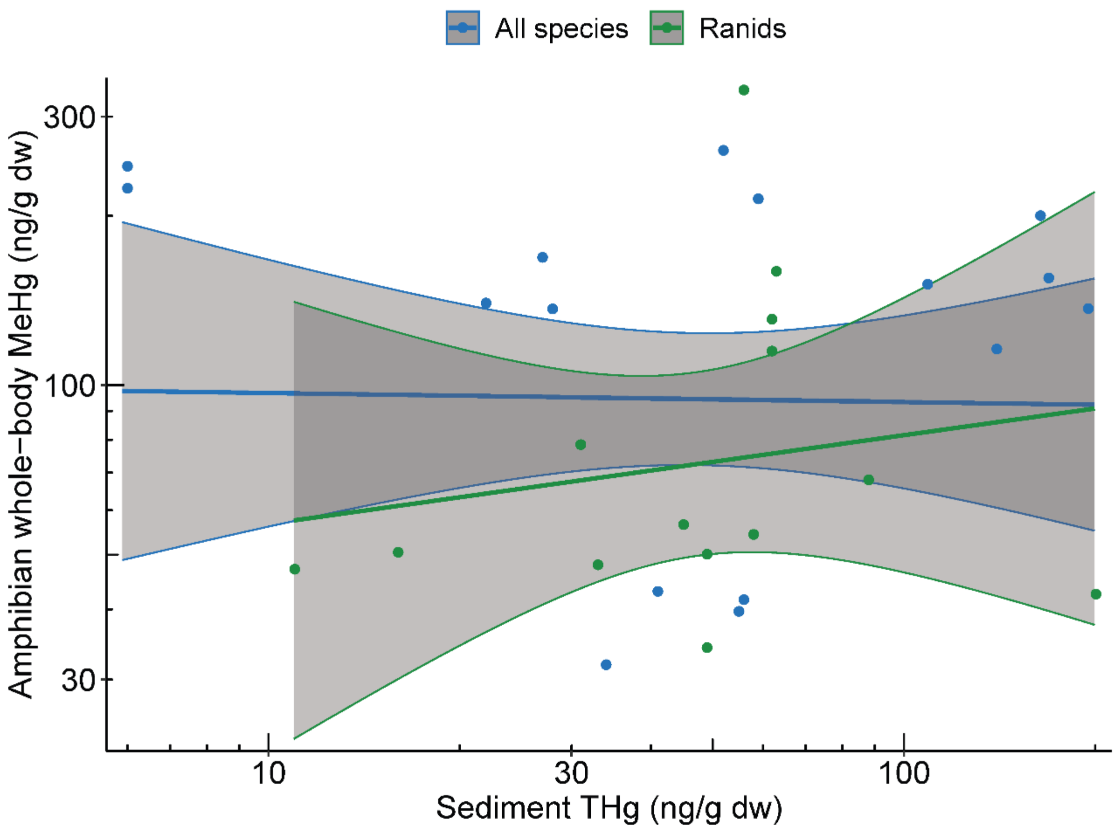

Supplementary Figure 4. Associations between mean (± 95% confidence interval) whole-body methylmercury (MeHg; ng/g dry weight [dw]) and sediment total mercury (THg; ng/g dw) from amphibians and sediment samples collected from the same locations in 2019. Both axes are log transformed and the all species estimate (blue line) contains all blue and green (ranid) points. The ranid estimate only includes green points. Neither line is a statistically significant correlation.

Equation S1. Regression equation describing relationship between amphibian toe clip and whole-body methylmercury (MeHg) concentrations (ng/g dw). Regression equations derived from: (CES data release).

$$log10\ whole\ body\ MeHg\ \left(\frac{ng}{g}dw\right) = \ 0.3255\ +\ 0.9511\ \times \mathrm{log10\ toe\ clip\ MeHg}\ \left(\frac{ng}{g}dw\right)$$

$$R^2 = 0.92, \mathrm{N} = 85$$

Equation S2. Regression equation describing relationship between amphibian tail clip and whole-body methylmercury (MeHg) concentrations (ng/g dw). Regression equations derived from: (CES data release).

$$log10\ whole\ body\ MeHg\ \left(\frac{ng}{g}dw\right) = \ 0.2092\ +\ 0.9882\ \times \mathrm{log10\ tail\ clip\ MeHg}\ \left(\frac{ng}{g}dw\right)$$

$$R^2 = 0.91, \mathrm{N} = 142$$
